# Vacuum-Assisted Delivery Complication Rates Based on Ultrasound-Estimated Fetal Weight

**DOI:** 10.3390/jcm11123480

**Published:** 2022-06-17

**Authors:** Hanoch Schreiber, Gal Cohen, Sivan Farladansky-Gershnabel, Maya Sharon-Weiner, Gil Shechter Maor, Tal Biron-Shental, Ofer Markovitch

**Affiliations:** 1 Department of Obstetrics and Gynecology, Meir Medical Center, Kfar Saba 4428164, Israel; galcwork@gmail.com (G.C.); sivangershnabel@gmail.com (S.F.-G.); swmaya@gmail.com (M.S.-W.); gilshec@gmail.com (G.S.M.); shentalt@inter.net.il (T.B.-S.); markovitch.ofer@clalit.org.il (O.M.); 2 Sackler School of Medicine, Tel Aviv University, Tel Aviv 6997801, Israel

**Keywords:** vacuum-assisted delivery, estimated fetal weight, shoulder dystocia, third- and fourth-degree perineal tears

## Abstract

This retrospective cohort study investigated the association between ultrasonographic estimated fetal weight (EFW) and adverse maternal and neonatal outcomes after vacuum-assisted delivery (VAD). It included women with singleton pregnancies at 34–41 weeks gestation, who underwent ultrasonographic pre-labor EFW and VAD in an academic institution, over 6 years. Adverse neonatal and maternal outcomes included shoulder dystocia, clavicular fracture, or third- and fourth-degree perineal tears. A receiver–operator characteristic curve was used to identify the optimal weight cut-off value to predict adverse outcomes. Fetuses above and below this point were compared. Multivariate analysis was used to control for factors that could lead to adverse outcomes. Eight-hundred and fifty women met the inclusion criteria and had sonographic EFW within two-weeks before delivery. Receiver–operator characteristic curve analysis found that ultrasonographic EFW 3666 g is the optimal threshold for adverse outcomes. Based on these results, outcomes were compared using EFW 3700 g. The average EFW in the ≥3700 g group (*n* = 220, 25.9%) was 3898 ± 154 g (average birthweight 3710 ± 324 g). In the group <3700 g (*n* = 630, 74.1%), average EFW was 3064 ± 411 g (birthweight 3120 ± 464 g). Shoulder dystocia and clavicular fractures were more frequent in the higher EFW group (6.4% and 2.3% vs. 1.6% and 0.5%, respectively; *p* < 0.05). Women in the ≥3700 g group experienced more third- and fourth-degree perineal tears (3.2% vs. 1%, *p* = 0.02). Multivariate logistic regression analysis found maternal age, diabetes and sonographic EFW ≥ 3700 g as independent risk-factors for adverse outcomes. Sonographic EFW ≥ 3700 g is an independent risk-factor for adverse outcomes in VAD. This should be considered when choosing the optimal mode of delivery.

## 1. Introduction

Recent decades have seen a trend of increasing birthweights in many developed countries [1,2,3,4]. Some of the reasons for this are the increased incidence of diabetes, advanced maternal age at pregnancy and higher pre-pregnancy BMI [3]. Increased awareness and various interventions may moderate this trend [5]; however, the percentage of fetal macrosomia is still high, in some places reaching 20% of deliveries [6]. Numerous maternal and neonatal complications have been associated with macrosomia, including low Apgar scores, shoulder dystocia, brachial plexus injuries, prolonged labor, instrumental delivery, perineal tears and postpartum hemorrhage [7,8,9,10,11,12].

In the UK, 10–15% of all women give birth via assisted vaginal delivery [13]. The most common indications are non-reassuring fetal heart rate and prolonged second stage of labor that, in many cases, are associated with increased birthweight [14]. As vacuum-assisted delivery (VAD) is an alternative to Cesarean section, its use should be considered according to the chance of success versus potential complications [15]. Several studies have investigated the association between increased fetal birthweights and maternal and fetal complications when VAD is performed. These studies demonstrated that with VAD, fetal macrosomia (defined as birthweight of 4000 g and above) is an important contributor to adverse outcomes, including lower Apgar scores, increased brachial plexus injury and subgaleal hematoma [6,16,17,18]. However, the decision to perform VAD is based on estimated fetal weight (EFW) and not on the actual birth weight, which is unknown at the time of the decision. Studies evaluating the rate of instrumental delivery complications in cases of suspected fetal macrosomia based on ultrasound EFW are lacking. Thus, the ACOG practice bulletin about operative vaginal birth recommends the “judicious use” of instrumental delivery when macrosomia is suspected [14]. Similarly, The Royal College of Obstetricians and Gynecologists recommends that, in cases of EFW greater than 4 kg or “a clinically big baby”, instrumental delivery should be conducted in a setting that allows immediate cesarean delivery and should be considered a trial of labor [13].

The objective of this study was to assess the association between ultrasonographic EFW and adverse outcomes during VAD.

## 2. Materials and Methods

This retrospective study included all women who had a singleton, vacuum-assisted delivery at term, from 2014 to 2020, in a single academic institution. As part of our departmental protocol, each patient admitted for delivery has a clinical or sonographic EFW performed at admission or within the previous two weeks. The EFW is documented in the electronic admission data by the physician before admission to the delivery room.

As the aim of the study was to examine the association between ultrasound-based fetal weight assessment and adverse outcomes, only participants with sonographic EFW were included. Hadlock’s formula was used for ultrasonographic EFW [19]. Maternal and neonatal outcomes were assessed. Adverse outcomes were defined as neonatal shoulder dystocia, clavicular fracture, or maternal third- or fourth-degree perineal tear. To characterize the complications associated with EFW and VAD only, cases of failed vacuum delivery were excluded.

Using receiver operating characteristic (ROC) curve analysis, we initially evaluated the sonographic EFW associated with significant VAD complications, which should therefore be considered a cut-off point for considering which mode of delivery to choose. The cut-off ultrasonographic EFW was found to be 3700 g.

Based on the ROC analysis, women were allocated into two groups: (1) high-ultrasonographic EFW (≥3700 g) or (2) low-ultrasonographic EFW (<3700 g).

We included patients with indication for prompt delivery and suitable conditions for VAD that did not have a previous attempt of instrumental delivery. Sylastic cup or a Kiwi Omnicup were used. Exclusion criteria were known significant fetal anomaly, antepartum fetal demise, multiple pregnancies, and gestational week earlier than 34.

The decision to perform VAD is made routinely by an attending gynecologist following complete evaluation of the indication, the fetal head station and position, and adequacy of the maternal pelvis. Each attempt at VAD is documented in detail, immediately after the intervention.

In our institution, the older classification for describing the fetal head station is used. In this classification system, the fetal head station is defined by thirds from −3 to +3 and not in centimeters [20]. However, in accordance with ACOG guidelines, VAD was performed only when the head station was at least 2 cm lower than the ischial spines.

All the records with diagnosis of VAD were reviewed, and data were obtained from the electronic medical records of the parturient and the neonate. The neonatal outcomes assessed included cephalohematoma, subgaleal hematoma, shoulder dystocia, clavicular fracture, Erb’s palsy, Apgar scores, umbilical cord pH and rate of NICU admissions. The maternal outcomes were third stage duration, amount of bleeding and the rate of third- and fourth-degree perineal tears.

A diagnosis of shoulder dystocia was recorded when additional obstetric maneuvers beyond gentle traction were needed to enable the delivery of the fetal shoulders.

### Statistical Analysis

Data were described as mean and standard deviation for continuous parameters and numbers and percentage for nominal data. *T*-test was performed to analyze continuous variables and chi-square for discrete categorical variables. ROC curve analysis was used to calculate the optimal threshold of sonographic EFW for adverse outcome. Multiple logistic regression was used to find variables that could explain the differences between the group of high fetal weight estimation (≥3700 g) and the group of low fetal weight estimation (<3700 g), after adjusting for confounders. Statistical analyses were performed using SPSS-25 software (IBM Corp., Armonk, NY, USA).

## 3. Results

From February 2014 through September 2020, there were 48,876 deliveries in our institution. Overall, 4079 women had VAD (8.3%). Among them, 850 met the inclusion criteria along with sonographic fetal weight estimation within two weeks before admission for delivery.

**Figure 1:**
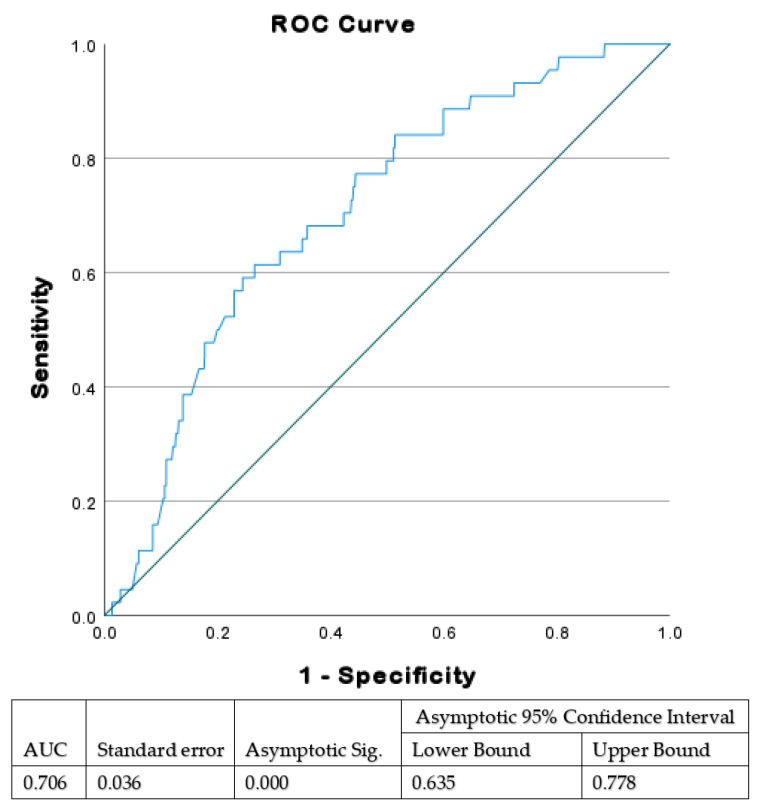
Receiver operating characteristic (ROC) analysis was used to identify a threshold of ultrasonographic EFW (3666 g) that was associated with adverse outcomes after VAD. Adverse outcomes were defined as shoulder dystocia, clavicular fracture, or third- and fourth-degree perineal tear. AUC, area under the curve.

Using a ROC curve (Figure 1), we found that ultrasonographic EFW of 3666 g is the optimal threshold value for adverse outcomes in fetuses delivered by VAD. Based on these results, outcomes were compared between groups divided according to EFW of 3700 g. Among the study population, the EFW was ≥3700 g in 220 (25.9%) and <3700 g in 630 (74.1%). The average EFW in the higher EFW group was 3898 ± 154 g with average birthweight of 3710 ± 324 g. In the lower EFW group, average EFW was 3064 ± 411 g with average birthweight of 3120 ± 464 g. Overall, the ultrasonographic EFWs were within 15% of actual birth weights in 761 women (89.5%), of which 193 (87.7%) were in the ≥3700 g group, and 568 (90.1%) were in the <3700 group (*p* = 0.31).

The baseline characteristics of the participants in both study groups are shown in Table 1. Several factors were associated with higher EFW, including gestational age and multiparity. There was tendency toward older maternal age, higher pre-pregnancy BMI and diabetes in the group of higher EFW, but this did not reach statistical significance.

The labor and delivery characteristics are shown in Table 2. Sylastic cup was used more frequently in the higher EFW group.

Maternal and neonatal outcomes are shown in Table 3. Shoulder dystocia and clavicular fracture were more common in the ≥3700 EFW group compared to the lower EFW group (6.4% and 2.3% vs. 1.6% and 0.5%, respectively; *p* < 0.05). Third- and fourth-degree perineal tears were more common in the higher EFW group (3.2% vs. 1%, respectively; *p* = 0.02). The overall rate of third- and fourth-degree perineal tears in our institution during the study period was 0.78%.

Outcomes were also analyzed by EFW cutoffs of 3900 g and 4000 g. Sonographic EFW > 3900 g and EFW > 4000 g were associated with Apgar 5 ≤ 7 (*p* = 0.05 and *p* = 0.008, respectively) and with higher maternal blood loss (426 mL vs. 346 mL, *p* = 0.005 and 498 mL vs. 347 mL, *p* < 0.001). No other differences in maternal or neonatal outcomes were found between groups.

A multivariate analysis model was used to control for potential factors for adverse outcomes. These included maternal age, diabetes, BMI, nulliparity, previous cesarean delivery, gestational age and the type of the cup used for the vacuum delivery. All adverse outcomes were found to be significantly more common in the higher EFW group. After controlling for all potential confounders, higher EFW (≥3700 g), maternal age and diabetes were found to be independent risk-factors for adverse outcomes (Table 4).

## 4. Discussion

The study results demonstrate that EFW ≥ 3700 g based on ultrasound is a significant risk factor for adverse maternal and neonatal outcomes. It is associated with higher rate of third- and fourth-degree perineal tears and with higher risk for shoulder dystocia and clavicular fracture.

According to our data, ultrasonographic EFWs were within 15% of actual birth weights in 761 women (89.5%). These results are in line with previous studies of EFW at term, with around 10% mean absolute error compared to the actual birthweight [21,22]. The most recent American College of Obstetricians and Gynecologists guidelines dealing with operative vaginal birth state: “There are no studies that evaluate the risk of complications with operative vaginal birth based on estimated fetal weight” [14]. As a result, there are no clear criteria for avoiding instrumental delivery given a high fetal weight estimation based on ultrasound. Indeed, high birthweight may result in prolonged second stage and is a well-known risk-factor for operative vaginal delivery. Various studies have shown that high birthweight is associate with increased risks for adverse outcomes during VAD [6,16,17,18]. The added risk of operative vaginal delivery with macrosomia is debatable. While some studies did not find increased risks with operative vaginal delivery compared to vaginal delivery, others found that among neonates with higher birthweights, complications were increased after VAD compared to vaginal delivery [6,16]. However, birthweight is not known prior to the birth, and when deciding the mode of delivery, the available data are limited. Therefore, the current study adds novel and practical data that may help in the decision-making process.

While it is difficult to predict which VAD will fail or have complications, a comprehensive assessment before performing a vacuum attempt can reduce the risks. Selecting the appropriate patient, the operator, and the setting is critical in this context. The data presented in the current study highlight the importance of ultrasound-based fetal weight estimation as an additional tool that should be used while evaluating risk-factors for adverse outcomes of VAD.

Our data suggest that despite its known limitations, prenatal fetal weight estimation has an important contribution in predicting complications of VAD. In addition, as information on estimated fetal weight and not birthweight is available before performing an instrumental delivery, the threshold for adverse outcomes should be defined based on estimated fetal weight pre-delivery.

The strengths of this study include the comprehensive information about the pregnancy and the delivery, allowing control for possible confounders. In addition, the data were collected from a single institution, VAD were performed using only non-metal vacuum cups and the same protocol. Only data about pregnancies with healthy fetuses based on prenatal care and only successful VAD were included. Therefore, adverse maternal or neonatal outcomes were not affected by known fetal malformations or any other procedures.

The limitations of the study are related to its retrospective design. Fetal weights were estimated by physicians and technicians with different skills. In addition, information regarding long-term outcomes was not available.

## 5. Conclusions

According to our data, ultrasonographic estimated fetal weight ≥3700 g is associated with adverse outcomes when VAD is conducted. However, it should be noted that although the difference was statistically significant, additional complications in the group with the higher EFW was low. Therefore, EFW should be evaluated as an additional component of the preliminary assessment before deciding on the mode of delivery. A large, prospective, double-blind, randomized, controlled trail should be conducted to examine whether high EFW is associated with increased risk for adverse outcomes. Depending on the results, it should be considered whether ultrasound EFW should be routinely performed.

## Figures and Tables

**Table 1:** Baseline characteristics in relation to sonographic EFW.

Characteristic	EFW ≥ 3700 g (*n* = 220)	EFW < 3700 g (*n* = 630)	*p*-Value
Maternal age (years ± SD)	31.0 ± 5.5	30.56 ± 5.2	0.250
Gestational age (weeks + days ± SD)	40 + 2 ± 6.6	39 + 3 ± 10.4	<0.001
Nulliparous, *n* (%)	132 (60)	439 (69.7)	0.011
VBAC, *n* (%)	26 (11.8)	102 (16.2)	0.118
Smoking, *n* (%)	8 (3.6)	40 (6.3)	0.133
Body mass index	26.05 ± 5.53	22.69 ± 9.2	0.113
Diabetes, *n* (%)	36 (16.4)	82 (13.0)	0.216
Chronic hypertension, *n* (%)	2 (0.9)	7 (1.1)	0.614
Pre-eclampsia, *n* (%)	1 (0.45)	21 (3.3)	0.02
EFW (g ± SD)	3898 ± 154	3064 ± 458	<0.001

SD, standard deviation; VBAC, vaginal birth after Cesarean section.

**Table 2:** Comparison of labor and delivery factors in relation to sonographic EFW.

Factor	EFW ≥ 3700 g (*n* = 220)	EFW < 3700 g (*n* = 630)	*p*-Value
Epidural, *n* (%)	199 (90.5)	571 (90.6)	0.93
Meconium-stained amniotic fluid, *n* (%)	43 (19.5)	97 (26.9)	0.457
Second stage duration (min ± SD)	144 ± 83	137 ± 82	0.3
Head position-OA, *n* (%)	172 (78.2)	487 (77.3)	0.952
Head station			0.151
S + 1, *n* (%)	125 (56.8)	333 (52.9)	
S + 2, *n* (%)	75 (34.1)	234 (37.1)	
S + 3, *n* (%)	1 (0.5)	14 (2.2)	
Missing data, *n* (%)	19 (8.6)	49 (7.8)	
Vacuum indication			0.95
NRFHR, *n* (%)	154 (70)	447 (70.9)	
Prolonged second stage, *n* (%)	49 (22.2)	134 (21.3)	
Other, *n* (%)	17 (7.8)	49 (7.8)	
Cup type			<0.001
Kiwi, *n* (%)	110 (52.9)	403 (67.3)	
Sylastic, *n* (%)	98 (47.1)	196 (32.7)	
Missing data, *n* (%)	12 (5.4)	31 (4.9)	
Cup detachment, *n* (%)	49 (22.2)	125 (19.8)	0.429
Episiotomy, *n* (%)	142 (64.5)	395 (62.7)	0.625
Birth weight, g ± SD	3710 ± 324	3120 ± 464	<0.001

SD, standard deviation; NRFHR, nonreassuring fetal heart rate.

**Table 3:** Maternal and neonatal outcomes in relation to sonographic estimated fetal weight.

Outcome	≥3700 g (*n* = 220)	<3700 g (*n* = 630)	*p*-Value
Apgar 1 ≤ 7, *n* (%)	36 (16.4)	73 (11.6)	0.068
Apgar 5 ≤ 7, *n* (%)	4 (1.8)	4 (0.6)	0.118
pH < 7.1, *n* (%)	0 (0)	4 (0.6)	0.577
pH < 7.15, *n* (%)	15 (7.8)	54 (9.8)	0.41
Third stage duration, min ± SD	9 ± 5	9 ± 6	0.74
Bleeding, mL ± SD	373 ± 241	347 ± 235	0.195
NICU, *n* (%)	6 (2.7)	19 (3)	0.827
Cephalohematoma, *n* (%)	6 (2.7)	20 (3.2)	0.74
Subgaleal hematoma, *n* (%)	18 (8.2)	36 (5.7)	0.196
Shoulder dystocia, *n* (%)	14 (6.4)	10 (1.6)	0.001
Clavicular fracture, *n* (%)	5 (2.3)	3 (0.5)	0.018
Erb’s palsy, *n* (%)	3 (1.3)	4 (0.6)	0.303
Third/fourth degree perineal tear, *n* (%)	7 (3.2)	6 (1.0)	0.02

SD, standard deviation.

**Table 4:** Multivariate logistic regression analysis for adverse outcome (defined if shoulder dystocia/clavicular fracture/third- or fourth-degree perineal tear occurred).

Variable	*p*-Value	Odds Ratio	95% Confidence Interval
Maternal age	0.019	0.921	0.860–0.986
BMI > 25	0.152	1.685	0.825–3.44
Diabetes	0.039	0.428	0.192–0.957
Nulliparity	0.99	0.994	0.365–2.706
VBAC	0.786	0.842	0.243–2.913
Gestational age	0.294	1.021	0.982–1.062
Cup type	0.297	0.715	0.381–1.343
EFW ≥ 3700	0.004	0.384	0.202–0.73

VBAC, vaginal birth after Cesarean section.

## Data Availability

Data will be made available from the corresponding author upon reasonable request.

## References

[1] Wang J., Moore D., Subramanian A., Cheng K.K., Toulis K.A., Qiu X., Saravanan P., Price M.J., Nirantharakumar K. (2018). Gestational dyslipidaemia and adverse birthweight outcomes: A systematic review and meta-analysis. Obes. Rev..

[2] Ghosh R.E., Berild J.D., Sterrantino A.F., Toledano M.B., Hansell A. (2018). Birth weight trends in England and Wales (1986–2012): Babies are getting heavier. Arch. Dis. Child.-Fetal Neonatal Ed..

[3] Goldstein R.F., Abell S.K., Ranasinha S., Misso M.L., Boyle J.A., Harrison C.L., Black M.H., Li N., Hu G., Corrado F. (2018). Gestational weight gain across continents and ethnicity: Systematic review and meta-analysis of maternal and infant outcomes in more than one million women. BMC Med..

[4] D’Souza R., Horyn I., Pavalagantharajah S., Zaffar N., Jacob C.-E. (2019). Maternal body mass index and pregnancy outcomes: A systematic review and metaanalysis. Am. J. Obstet. Gynecol. MFM.

[5] Morisaki N., Esplin M.S., Varner M.W., Henry E., Oken E. (2013). Declines in Birth Weight and Fetal Growth Independent of Gestational Length. Obstet. Gynecol..

[6] Åberg K., Norman M., Pettersson K., Ekéus C. (2016). Vacuum extraction in fetal macrosomia and risk of neonatal complications: A population-based cohort study. Acta Obstet. Gynecol. Scand..

[7] Temerinac D., Chen X., Sütterlin M., Kehl S. (2014). Influence of fetal birth weight on perinatal outcome in planned vaginal births. Arch. Gynecol. Obstet..

[8] Bjørstad A.R., Irgens-Hansen K., Daltveit A.K., Irgens L.M. (2010). Macrosomia: Mode of delivery and pregnancy outcome. Acta Obstet. Gynecol. Scand..

[9] Boulet S., Alexander G.R., Salihu H.M., Pass M. (2003). Macrosomic births in the united states: Determinants, outcomes, and proposed grades of risk. Am. J. Obstet. Gynecol..

[10] Herzberg S., Kabiri D., Mordechai T., Yahya R.H., Chill H., Levitt L., Amsalem H., Ezra Y. (2017). Fetal macrosomia as a risk factor for shoulder dystocia during vacuum extraction. J. Matern. Fetal Neonatal Med..

[11] Beta J., Khan N., Khalil A., Fiolna M., Ramadan G., Akolekar R. (2019). Maternal and neonatal complications of fetal macrosomia: Systematic review and meta-analysis. Ultrasound Obstet. Gynecol..

[12] Segal D., Baumfeld Y., Yahav L., Yohay D., Geva Y., Press F., Weintraub A.Y. (2018). Risk factors for obstetric anal sphincter injuries (OASIS) during vacuum extraction delivery in a university affiliated maternity hospital. J. Matern. Fetal Neonatal Med..

[13] Murphy D.J., Strachan B.K., Bahl R. (2020). the Royal College of Obstetricians and Gynaecologists Assisted Vaginal Birth. BJOG Int. J. Obstet. Gynaecol..

[14] American College of Obstetricians and Gynecologists’ Committee on Practice Bulletins—Obstetrics (2020). Operative vaginal birth: ACOG practice bulletin, number 219. Obstet. Gynecol..

[15] Yüksel I.T., Çetin B.A., Şenol G., Akça A., Aydın A. (2020). Comparison of Cesarean Sections Performed in the Second Stage of Labor and Vacuum-assisted Vaginal Delivery. Eur. Arch. Med. Res..

[16] Kolderup L.B., Laros R.K., Musci T.J. (1997). Incidence of persistent birth injury in macrosomic infants: Association with mode of delivery. Am. J. Obstet. Gynecol..

[17] Henriksen T. (2008). The macrosomic fetus: A challenge in current obstetrics. Acta Obstet. Gynecol. Scand..

[18] Ekéus C., Högberg U., Norman M. (2014). Vacuum assisted birth and risk for cerebral complications in term newborn infants: A population-based cohort study. BMC Pregnancy Childbirth.

[19] Hadlock F.P., Harrist R.B., Sharman R.S., Deter R.L., Park S.K. (1985). Estimation of fetal weight with the use of head, body, and femur measurements—A prospective study. Am. J. Obstet. Gynecol..

[20] Gabbe S.G., Niebyl J.R., Simpson J.L. (2007). Obstetrics: Normal and Problem Pregnancies.

[21] Oliver M., McNally G., Leader L. (2013). Accuracy of sonographic prediction of birth weight. Aust. N. Z. J. Obstet. Gynaecol..

[22] Sekar R., Duncombe G., Khatun M., Barrett H.L. (2015). A prospective pilot study in assessing the accuracy of ultrasound estimated fetal weight prior to delivery. Aust. N. Z. J. Obstet. Gynaecol..

